# Supplementary material for: Thrombus characteristics evaluated by acute optical coherence tomography in ST elevation myocardial Infarction
Source: PLoS One. 2022 Apr 11;17(4):e0266634. doi: 10.1371/journal.pone.0266634 (PMC9000063; doi:10.1371/journal.pone.0266634)
Supplement: S1 Text — (DOCX) [file pone.0266634.s002.docx]

**Project summary**

Patients presenting with acute ST elevation myocardial (STEMI) infarct urgently need revascularization. Standard of care is establishing bloodflow through the coronary vessels using thrombus aspiration catheter, and securing the result by using a metallic drug eluting stent. New kinds of non-metallic bioresorbable scaffolds are now available. They have however challenges in structural strength.

We want to compare the new bioresorbable scaffold with traditional metallic stents in a prospective, randomized, non-blinded, multicenter study in 120 STEMI patients. We will use optical coherence tomography to evaluate the results after 12 months.

We also want to see if modern multislice computed tomography can give useful information in the follow-up of stented coronary arteries after 12 and 24 months.

We are also analyzing thrombus aspirates for markers for stent performance.

**General Information**

Title: **Performance of bioresorbarble scaffold in primary percutaneous intervention of ST elevation myocardial infarct.**

Investigators:

Erlend Eriksenᵃᵇ (Project leader)

Øyvind Bleieᵃᵇ

Jon Herstadᵃᵇ

Niels Holm, Skejbyᶜ

Allard C van der Wal ᵈ

Christian Eekͤ

Thor Styrk Trovikᶠ

Vegard Tusethᵇ

Erik Packerᵇ

Jan Erik Nordrehaugᵃᵇ

ᵃHaukeland University Hospital, Helse Bergen, Dep of heart disease, Norway

ᵇUniversity of Bergen

ᶜAarhus Universitetshospital, Skejby, Danmark

ᵈAcademic Medical Center/University of Amsterdam, Department of Pathology

ͤOslo Universitetssykehus, Rikshospitalet

ᶠUniversitetssykehuset i Nord-Norge

Abbreviations

STEMI ST elevation myocardial infarct

ESC European Society of Cardiology

DES Drug eluting stent

BVS Biodegradable Vascular Scaffold

OCT Optical coherence tomography

DAPT Dual antiplatelet therapy

BVS Bioresorbable Scaffold

IVUS Intravascular ultrasound

GFR Glomerulus filtration rate

BP Blood pressure

MLA Minimum luminal area

PLLA Poly-L-Lactide

PDLLA Poly-D,L-Lactide

TLR Target lesion revascularization

TVR Target vessel revascularization

CCS Canadian Cardiology Society

MI Myocardial infarct

MSCT Multislice computer tomography

**1 Rationale and background information**

- 1. **Background and current standard of care**

In patients presenting with myocardial infarct with ST elevation, Primary percutaneous coronary intervention (PCI) is the preferred treatment (ESC guidelines recommendation I A). Stenting has I A recommendation, and preferably with drug eluting stents (DES) in patients who are likely to be compliant to dual antiplatelet therapy (DAPT) and are not at an increased bleeding risk (II A recommendation). Thrombus aspiration with specialized catheter has an equally strong II A recommendation[1]. Thrombus aspiration followed by direct stenting without any further balloon dilatation has been shown to give better 1 year survival[2-6].

- 1. **New stent platforms**

Metallic drug eluting stents are being challenged by new stents made from biodegradable platforms, or BVS (Biodegradable Vascular Scaffold). The Absorb BVS (Abbott Vascular, Santa Clara, CA) is the first of these being commercially available in Norway. The second-generation Absorb BVS is a balloon-expandable device consisting of a polymer backbone of poly-l-lactide (PLLA) coated with a thin layer of a 1:1 mixture of an amorphous matrix of poly-d, l-lactide (PDLLA) polymer and 100 μg/cm^2^ of the antiproliferative drug everolimus. Two platinum markers located at each Absorb BVS edge allow for accurate visualization of the radiolucent Absorb BVS during angiography or other imaging modalities. The PDLLA controls the release of everolimus, 80% of which is eluted within the first 30 days. Both PLLA and PDLLA are fully bioresorbable. The polymers are degraded via hydrolysis of the ester bonds, and the resulting lactate and its oligomers are quickly transformed to pyruvate and metabolized in the Krebs energy cycle. Small particles, less than 2 μm in diameter, have also been shown to be phagocytized and degraded by macrophages. According to preclinical studies, the time for complete bioresorption of the polymer backbone is 2 to 3 years It has been proven to be safe and non-inferior to metallic everolimus stents in the ABSORB II, kohort B trial [7, 8]. The bioresorbable scaffold has obvious advantages over metallic, as it will not hinder any future revascularization. However there are concerns regarding the strength of the scaffold. It is therefore recommended with more rigorous lesion preparation, using balloons for predilatation. Currently only one small study has been published studying specifically BVS in STEMI patients. This study is not randomized and does not address the issue of predilatation[9].

**1.2 Imaging OCT**

OCT (optical coherence tomography) is emerging as a useful diagnostic tool in the treatment of coronary disease. The prospects for use in plaque assessment, thrombus characterization, strut apposition, acute damage after stenting and no-reflow situations are promising for clinical use in the acute setting in the cath lab. Furthermore, OCT assessment of tissue coverage and restenotic tissue characterization are invaluable tools in follow up studies in patients with coronary stents. It also gives a detailed image of the surface and immediate sub intimal layers with details that exceeds intravascular ultrasound (IVUS) with about tenfold better resolution (10-15 µm vs. 100-200µm)[10, 11]. Consensus standards for the use of OCT gives at firm base to standardize further OCT based investigations[12]. OCT is thus a superior tool in evaluating percutaneous coronary interventions techniques[13, 14]. OCT will also emerge as a valuable clinical tool in the acute setting in the cath lab. Introduction of OCT to our lab through a study like this, will ensure that the operators at our lab master this tool and its interpretation[15].

- 1. **Imaging MSCT**

Multislice computed tomography (MCST) is a non-invasiv method of imaging coronary arteries. It is however currently not specific enough to evaluate arteries with previous coronary stent. By performing av MSCT at 12 months and 24 months follow up, we will both extend the follow up period by non-invasive method and by comparing to OCT and angiography, hopefully improve coronary diagnostics by MSCT imaging. The results will be published in substudy papers. Details are described in separate protocol.

- 1. **Thrombus analysis**

**Methods**.

Aspirate from aspiration catheter Export Advance (Medtronic) will be filtered through 40 µm filter basket before fixation  in 4% buffered formalin. Aspirate will be fixed in formalin for at least 24 hrs before shipment to Amsterdam Medical Center for further embedding in paraffin and to be cut in 5µm slices and stained with:

1. Haematoxylin and Eosin (HE)  and elastic  van Gieson (EvG) stains respectively[[16-18](#_ENREF_16)] for conventional histomorphological evaluation.
2. I mmunohistochemical staining with SMA-1 antibody (smooth muscle cells), ant CD31antibody (endothelial cells and platelets) and anti fibrin.

**Analysis of tissue sections**

1. Sample size: aspired materials  will be measured in the tissue sections in mm2 morphometrically.
2. Histological compostion of aspirate: presence of thrombus material, plaque material or both in each aspirate will be recorded. Thrombus composition will evaluated semiquantitatively on presence and extent of erythrocyte content (HE), plateletcontent (CD31 immunostain), fibrin content (Fbrin immunostain), and granulocytes.
3. Thrombus age will be measured analytically according to previously published definitions of thrombus age:
4. Fresh (upto 1day): Composed of layered patterns of platelets, fibrin, erythrocytes and intact granulocytes.
5. Lytic thrombus (1-5 days): Characterized by areas of colliquation necrosis and/or karyorrhexis of granulocytes, presence of NET’s,
6. Organized thrombus (> 5 days): Ingrowth of smooth muscle cells, with or without depositions of connective tissue and capillary vessel ingrowth. (conventional-  and immunostains)

**Rationale**

The pathological analysis will be compared to pre-,post- PCI and follow-up OCT readings to establish if composition, size and age predicts residual thrombus pre PCI as measured stent result after PCI and primary and primary endpoint at follow-up as measured by OCT. These results will be published in a separate substudy paper.

- 1. **Study goals and objectives**

Study goal is to compare the performance of the Absorb bioresorbable scaffold with a metallic drug eltuting stent in the STEMI patient.

Hypothesis is that a bioresorbable vascular scaffold (BVS) is non-inferior to standard of care, eg. direct stenting with metallic DES, using direct stenting after thrombus aspiration.

**2.0 Study design**

The study is a prospective, randomized, controlled, non-blinded, multicenter study comparing metallic drug eluting stent with bioresorbable scaffold in STEMI patients. Primary endpoint Coronary Stent Healing Index, see 2.2.6 for definition.

**2.2 Patients**

We propose to include 120 patients presenting with STEMI with less than 12 hrs. history.

**2.2.1 Inclusion criteria are:**

1. History of chest pain < 12 hrs
2. ST elevation of ≥ 2 mm in ≥2 contigous precordial leads (V1-V6), and/or ≥ 1 mm in ≥ 2 contiguous standard leads (I, II, III, aVf, aVr,aVl).
3. Single vessel disease/ No other lesions expected to need recasularization
4. Clinical decision to treat with primary PCI
5. > 18 years
6. Oral informed consent

**2.2.3 Exclusion criteria:**

1. Contraindications to long term DAPT
2. Known kidney failure with GFR < 45
3. Cardiac arrest or severe cardiogenic shock (Persistent BP <90 mmHg, despite adequate treatment)
4. Other severe illness with life expectancy of less than 12 months (eg. malignancy, severe malnutrition, degenerative disease)

**2.2.4 Procedural contraindications:**

1. Heavy calcification, tortuous vessel or large side branch (> 2,5 mm) at culprit lesion.
2. TIMI 0-1 flow after aspiration
3. Unable to advance thrombus aspiration catheter

**2.2.5 Schedule of event registration**

Clinical events will be note at index procedure. Participants will be contacted by telephone after 3 and 6 months, and clinical control as outpatient at 12 months with MCST and echocardiography.

**2.2.6 Primary endpoint**

Coronary Stent Healing Index (cumulated):

1. Uncovered struts: 2% =1 – 5% =2 – 10% =3 - 15% =4 – 20% =5 – 25% =6 – 30% =7 – 35% =8 – 40% =9

2)    Uncovered struts in fornt of side branch on acquired or persistent malapposed struts. 10% =1 – 20% =2 – 30% =3 etc… til 100%=10

3)     Persistent malapposition: ≥2 nabo struts længde mindst 1 mm =1 ; ≥2mm=3 ; ≥3 mm = 3

4)     Acquired malapposition: ≥2 adjacent struts of at least 1 mm length  =2 ; ≥2mm=4 ; ≥3 mm = 6

5)     Neointimal thickness in one frame >200 =1 - >300 =2  - >400 =3 or diameter stenosis >50% =4 - > 75% =5

6)      Cumulated extra stent lumen increase in match cross sectional analysis: (gns. areal mål): ≥0.2mm2 =1 ; ≥0.4 mm2 = 2; ≥0.6mm2=3 ; ≥0.8 mm2 = 4 ;  ≥1.0 mm2=5 ; ≥1.2 mm2 = 6

**2.2.7 Secondary endpoints**

**2.2.7.1**

OCT endpoints

1. Area stenosis
2. Lumen late loss
3. Crushed stent segments
4. Malapposition of stent struts
5. Minimum expansion of the stents expressed as absolute area and percentage of the closest reference area,
6. Vessel ostial stent area (acute and at FU)
7. Thrombus burden

**2.2.7.2**

Angiographic endpoints

1.TIMI flow pre and post PCI,

2.Blush grade

3.Thrombus burden

4. Angiographic complications

5. Contrast use

6. Procedure time

7. Radiation skin dose

**2.2.7.3**

Clinical endpoints

Index admission:

1.total death

2. Cardiac death

3. Non-index procedure myocardial infarction

4. Stent thrombosis (definite and probable)

5. TLR and TVR

6. non-TVR

Follow up:

1. Total death

2. Cardiac death

3. Non-index procedure myocardial infarction

4. Stent thrombosis (definite and probarble)

5. TLR and TVR

6. non-TVR

7. CCS angina class

8. Vascular cerebral events

9. Admissions for congestive heart failure or arrhythmia

**2.2.8 Endpoints definitions: OCT**

Follows definitions from the “Consensus Standards for Acquisition, Measurement, and Reporting of Intravascular Optical Coherence Tomography Studies” paper by Tearney et al. JACC 2012

- - Segments to follow definitions in coming consensus standards
- Quantitative measurements
  - Segments to follow definitions in coming consensus standards
- Primary endpoint is Coronary Healing Score, see 2.2.6 for definition.

**2.2.9 Defination of endpoints (clinical)**

#### 2.2.9.1 All cause death (total death)

Total death encompasses cardiac death and other fatal categories, which include cerebrovascular death, death from other cardiovascular disease (i.e. pulmonary embolism, dissection aortic aneurysm will be included in this category), death from malignant disease, death from suicide, violence or accident, or death from other reasons.

#### 2.2.9.2 Cardiac death

Cardiac death encompasses coronary heart disease death including fatal myocardial infarction, sudden cardiac death including fatal arrhythmias and cardiac arrest without successful resuscitation, death from heart failure including cardiogenic shock, and death related to a cardiac procedure or surgery within 28 days from the procedure.

#### 2.2.9.3 Non-procedure related myocardial infarction

The term myocardial infarction should be used when there is evidence of myocardial necrosis in a clinical setting consistent with myocardial ischemia. Under these conditions any one of the following criteria meets the diagnosis for myocardial infarction (11):

1) Detection of rise and/or fall of preferably troponin T with at least one value above the 99th percentile of the upper reference limit (URL) together with evidence of myocardial ischemia with at least one of the following (MI types 1 or 2):

a. Symptoms of ischemia

b. ECG changes indicative of new ischemia (new ST-T changes or new LBBB)

c. Development of pathological Q waves in the ECG

d. Imaging evidence of new loss of viable myocardium or new regional wall motion abnormality

2) Sudden, unexpected cardiac death, involving cardiac arrest, often with symptoms suggestive of myocardial ischemia, and accompanied by presumably new ST elevation, or new LBBB, and/or evidence of fresh thrombus by coronary angiography and/or at autopsy, but death occurring before blood samples could be obtained, or at a time before the appearance of cardiac biomarkers in the blood (MI type 3).

3) Pathological findings of an acute myocardial infarction.

#### 2.2.9.4 Procedure related biochemical release of markers (Myocardial Infarction related to the Index Procedure)

The following biomarkers may be used in the study: CK-MB mass and/or Troponin-T/-I. For percutaneous coronary interventions (PCI) in patients with normal baseline biomarker values, elevations of cardiac biomarkers greater than 5 X 99th percentile URL are designated as defining PCI-related myocardial infarction (MI type 4a). If cardiac troponin is elevated before the procedure and not stable for at least two samples 6 hours apart, there are insufficient data to recommend biomarker criteria for the diagnosis of peri-procedural myocardial infarction. If the values are stable or falling, criteria for re infarction by further measurement of biomarkers can be applied; that is 20% or more increase of the value in the second sample after the procedure. This value should also exceed the 99th percentile URL .

#### 2.2.9.5 Target lesion revascularization

Coronary artery bypass grafting with grafting or PCI of index lesion.

#### 2.2.9.6 Target vessel revascularization

Coronary artery bypass grafting with grafting or PCI of index vessel.

#### 2.2.9.7 Stent thrombosis

Stent thrombosis is recognized when documented by angiography and/or autopsy and when meeting the criteria for spontaneous myocardial infarction occurring in the territory of the treated vessel (11). Stent thrombosis are categorized as acute, sub-acute, late and very late and as definite, probable and possible according to the ARC-criteria (12).

**2.2.10 Biochemical analysis**

**2.2.10.1** Creatinin, hemoglobin, Troponin T will be analyzed during index procedure post procedure and at 12 months follow up. ProBNP will be analyzed at 12 months follow-up

**2.2.10.2** Plasma will be drawn immediately after the procedure and frozen in a bio bank for later analysis.

**2.2.10.3** Blood from target vessel will be drawn for analysis of markers of myocardial necrosis from thrombus aspiration catheter.

**2.1.10.4** Visibible thrombus aspirates will be sent for analysis at Amsterdam Medical Center.

**2.2.11** **Optical coherence tomography core lab**

### OCT off line analysis is performed at the Interventional Imaging Core Laboratories at the Department of Cardiology Aarhus University Hospital, Skejby, Aarhus, Denmark. All files to be transferred on CD´s or DVD´s in RAW format.

### 2.2.12 OCT acquisition technique

### OPTIS St. Jude Medical OCT system will be used. Pre PCI: (after predilatation) OCT imaging catheters will be inserted and advanced to a position distal to the lesion and acquisition of the lesion will be performed at 36 mm/s (OPTIS). Final result acquisition: the OCT imaging catheters will be inserted and advanced to a position with acquisition start at least 5 mm distal to the distal stent edge. Pullback speed 20 mm/sec covering the. If target segment is longer than 53 mm then an additional pullback is performed covering entire target area. Final result is acquired by OCT. Flushing is performed by automated injector or manual injection. Iomerone contrast will be used for flushing. In case of difficult flushing, a larger guiding catheter or a more co-axial position of the guiding catheter is recommended. OCT files from baseline, follow-up and events (pre-intervention) should be submitted in RAW format to the Interventional Imaging Core Laboratory at Aarhus University Hospital via the study organization. Flushing for 3 seconds of the OCT imaging wire immediately before each pullback is mandatory

Careful evaluation of acquired pullback before finalizing procedure:

- Blood in catheter?
- Insufficiently flushed vessel?
- Target area acquired?
- SB ostium visible? (excess wire shadow?)

REPEAT PULLBACK IF QUALITY IS INSUFFICIENT

**2.3 Treatment step-by-step**

Before arrival et cath lab, loading with acetylic salisylic acid and one of the antiplatelet agents clopigrogel/pragusgrel/ticagrelor is mandatory. Heparin or bivalirudin at start of procedure. After angiography and confirmation of presence of thrombus GpIIb/IIIa inhibitors are administered at operators discretion.

After thrombus aspiration with TIMI flow 2 or more the patient will be asked to participate in the study. Oral consent witnessed by staff non-affiliated with the study will be obtained.

OCT scan will be performed after 0.2 mg intracoronary nitroglycerine for sizing and lesion evaluation. The patient is then randomized into one of two arms in a 1:1 distribution:

1. Direct stenting with everolimus eluting stent
2. Direct stenting with bioresorbable stent

Direct stenting is the preferred method of implantation to keep procedure as short and simple as possible. Predilatation will not considered protocol violation if deemed necessary by operator. After stenting and post dilation at operator’s discretion, a second OCT will be performed. Any further post dilatation will be performed at operator’s discretion. A final OCT will be done if further postdilatation is performed. 0.2 mg intracoronary nitroglycerine is administered before each OCT scan.

Patient will be asked to sign an informed consent after the procedure (<24 hrs).

**2.4 Antiplatelet therapy**

12 months dobble platelet therapy (DAPT) with acetylic salisylic acid and either ticagrelor or prasugrel.

**2.5 Multi vessel disease**

This is a single vessel disease study. Should other lesions need treatment at index hospitalization, the use of everolimus DES is recommended.

**2.6 Angiographic and OCT follow-up**

After 12 (± 1) months a diagnostic angiography by femoral or radial route will be performed and the projections used at the index angiography will be repeated after 0.2 mg intracoronary nitroglycerine. OCT acquisition is performed by 6F guiding catheter or larger.

In case of angiographic stenosis, revascularization should only be performed if FFR<0.8 or other objective proof of ischemia has been acquired.

The 12 months follow-up angiograms and the OCT runs will be stored on CDs and sent by mail to the Core Laboratory for Interventional Imaging angiographic core laboratories.

In case of any target lesion revascularization before 12 months a coronary angiogram and OCT should be performed.

**3.0 Data management and statistical analysis**

**3.1 Primary endpoint**

The primary endpoint in the two groups will be analyzed on patient level using paired t-test. A p-value considered indicating significance will be 5%.

**3.2 Secondary endpoints and other variables**

Differences in categorical variables between the two groups will be analyzed using the chi-square test or Fisher´s exact test. Continuous variables will be analyzed using independent sample t-test and Mann-Whitney U-test, and time to event data will be analyzed using the Kaplan-Meier method and the log-rank test. OCT and angiographic parameters will be evaluated by Spearman correlation analysis. Binary logistic-regression analysis will be performed to identify independent predictors of difference in outcome. Pre and post PCI OCT of vessel size, lesion severity, lesion length. All p-values are two-sided. Level of significance is 5%. The analysis is performed on an intention to treat basis and by actual treatment.

**3.3 Sample size calculation**

The Coronary Healing Score has not previously been validated in studies. The sample size calculation is therefore done using a Minimum flow Area (MinFA), the endpoint used in the TROFI trial, also investigating STEMI patients with OCT at follow up.

Following the assumptions of a Non-Inferiority design the sample size calculations of this study were based on pooled data of Cardialysis database from multiple trials in acute coronary syndrome where the MinFA was 4.95 + 1.39 mm2. The trial is powered for testing of noninferiority for the primary angiographic endpoint (Minimum Flow Area). In order to be able to reject the null hypothesis that standard treatment with DES produces a MinFA of at least 0.72mm2 better then the experimentaly treated group (BVS) requires a sample size of 54 patients per treatment group (alpha = 0.05 , power = 0.85 , predefined noninferiority margin = 0.72 mm2).

**3.4 Analysis of the population**

The primary endpoint will be analyzed according to the intention-to-treat principle.

Per protocol analysis of actual treatment will be performed.

**4. Randomization and data management**

**4.1 Randomization**

The participants will be randomized using online randomization tool provided by TrialPartner.

**4.2 Data management**

The study is approved by the Norwegian regional ethics committee (MREK) and “personvermombudet” from “Datatilsynet”.

The OCT images will be coded and all patient identification will be removed. Images will be stored in a digital storage unit. The core lab will not be blinded to which treatment arm the images belong, as the stents have different appearance on OCT images. The analysis will follow the “Consensus standards for Acquisition, Measurement, and Reporting in Intravascular Optical Coherence Tomography Studies”.

Data will be stored and analyzed according to Ethical committee guidelines.

**5 Quality assurance**

Clinical Events Comitee (CEC) by Per Mølstad (leader) and Olaf Rødevand (Feiring-LHL klinikkene) will assess all Serious Adverse Events (SAE), the primary endpoint and the clinical secondary endpoints.

To be reported as SAE are unexpected clinical happenings leading to:

Life threatening incidences

Prolonged hospitalization

Persistant or significant disability

In addition:

All death

All incidences of contrast nephropathy

All incidences of loss of flow in coronary vessels after stenting (no reflow and side branch occlusions).

All cases of stent failure, eg stent thrombosis, stent fracture, loss of stent.

All bailout stenting with DES in the BVS group.

Expected clinical events not related to the stents will not be reported, such as congestive heart failure (including shock), arrhythmias, rehospitalization without evidence of restenosis or stent thrombosis.

An independent Data Safety Monitoring Board (DSMB) committee led by Jose Henriques (Amsterdam Medical Center) will evaluate safety after inclusion of 40 patients within 30 days of inclusion.

**6 Expected outcome of the study**

We expect to find no difference in the minimal flow area, or the secondary endpoints of MACE and stent failure.

**7 Dissemination of result and publication policy**

The results will be published in an international journal for cardiac intervention.

**8 Duration of the project**

It is feasible to include 120 patients in our study within a 12 months period

**9 Problems anticipated**

Stent under expansion and stent fracture in the Absorb group is a potential problem. If technical problems with BVS or stentfracture/damage occurs, implanting DES (Xience) will be used as a “bailout” strategy.

**10 Project management**

The Steering Committee is the main policy and decision-making committee of the study and has final responsibility for the scientific conduct of the study.

The specific tasks of the Steering committee is to (1) approve the study protocol; (2) approve amendments to the protocol; (3) establish the organizational structure; (4) select the members of the various committees; (5) review the activities of the study committees and change these committees if found necessary, (6) act upon recommendations of the DSMB; (7) approve study reports and papers for publication.

The Steering Committee meets at the request of the Principal Investigators and/or the DSMB.

The Steering Committee constitutes: Erlend Eriksen, Øyvind Bleie, Vegard Tuseth, Jon Herstad, Karel Kuiper and Niels Holm.

**11 Ethics**

**11.1 Ethical conduct of the study**

The study will be conducted in accordance with the protocol, applicable regulatory requirements and the ethical principles of the Declaration of Helsinki as adopted by the 18th World Medical Assembly in Helsinki, Finland in 1964 and subsequent versions.

It is the responsibility of the investigator to obtain approval of the study protocol/protocol amendments, the patient information and the informed consent form from the Ethical Committee for all patients included in the study.

**11.2 Risk, side effects, advantages and disadvantages in participating in the study**

The extra risk for the patient during the index procedure is minimal as the both OCT and the stents used in both arms are considered safe. The primary procedure time will be about 10-20 minutes longer.The BVS has lower structural strength, but has the advantage of leaving behind now foreign object in the body after resorbtion.

The extra risk is primarily related to the control angiography. Major adverse effects are related to bleeding and hematoma at access point. More serious events such as cerebral insult and damage of coronary vessels are very rare (< 0, 5%), but a potential risk for the patient. The radiation required for a single angiogrammay cause a small increase in risk of developing cancer from 25,00% to 25.01%.

OCT does require an infusion of about 15-20 ml of contrast medium. This is a small dose, and should not pose any threat of contrast induced nephropathy for the individual with a GFR > 45. The risk is < 0,1%. Wire manipulation gives some risk for wire induced damage to the vessel, e.g. dissection and perforation, but is rare < 1%. With FD OCT patients in a minority of cases experience transient chest pain oppression during acquisition. T wave abnormalities have been observed (10%), but no major vascular complications (1).

The follow up by phone and one clinical visit cause some inconvenience for the participants.

Given the potentially benefit of the study, the risks involved are small and can be ethically justified.

**11.3 Patient information and consent**

The study group has opted for an initial oral consent before inclusion. In a setting of an acute STEMI, a delay in the revascularization of an occluded vessel will cause further myocardial damage. It will also not be clear if the patient is eligible for the study until an angiography has been done and flow in the vessel has been established.

Acquiring a written consent during the procedure will be impossible, as most procedures are done with access through the right wrist.

Acquiring written consent post procedure allows the investigator the time to fully explain the study in detail and sufficient time for the participant for consideration. A third party may also be present or consulted if preferred.

In our opinion, this is acceptable, because the primary risk and inconvenience is during follow up. The participants can withdraw their consent at any stage, including not going through with follow up angio.

**11.4 Withdrawal**

A participant will be considered withdrawn from the study if judged by the investigator as:

Not eligible

Protocol violation

Participants decides to withdraw consent

If participant withdraws consent, data collected up until withdrawal will be kept in the study if participant agrees.

Participants who does not return for follow-up will be described as “lost to follow up”.

**12 Biological material**

Plasma from blood drawn from aspiration catheter and peripheral vein will be frozen and kept for later analysis for markers of myocardial ischemia and necrosis. Thrombus from aspiration catheter will be fixed in formalin and sent to Amsterdam Medical Center for further analysis.

**13 Study plan**

Launch of study is expected by July 2014. Inclusion of patients is expected to last until1st quarter of 2016. Last follow up is then 1^st^ quarter of 2017.

**14 Economy**

Investigator has received an unrestricted grant from ST Jude Medical to cover cost of OCT core lab.

The individual hospital departments will cover cost of stents, OCT and expenses anticipated with follow up. Department of Heart Disease at Haukeland University Hospital will also have a study- and image- technician available to assist.

Investigator will apply for a Helse Vest PhD grant to cover labor cost of investigator and assisting staff.

The study has potential commercial interest for Abbot Vascular Solutions.

**14 Publication and ownership of data**

All information and data generated in association with this study will be held in strict confidence and remains the sole property of Department of Heart Disease, Haukeland University Hospital, Helse-Bergen HF.

**References**:

1. Task Force on Myocardial Revascularization of the European Society of, C., et al., *Guidelines on myocardial revascularization.* Eur Heart J, 2010. **31**(20): p. 2501-55.

2. Vlaar, P.J., et al., *Cardiac death and reinfarction after 1 year in the Thrombus Aspiration during Percutaneous coronary intervention in Acute myocardial infarction Study (TAPAS): a 1-year follow-up study.* Lancet, 2008. **371**(9628): p. 1915-20.

3. De Luca, G., et al., *Adjunctive mechanical devices to prevent distal embolization in patients undergoing mechanical revascularization for acute myocardial infarction: a meta-analysis of randomized trials.* Am Heart J, 2007. **153**(3): p. 343-53.

4. Burzotta, F., et al., *Feasibility of sequential thrombus aspiration and filter distal protection in the management of very high thrombus burden lesions.* J Invasive Cardiol, 2007. **19**(8): p. 317-23.

5. Dudek, D., et al., *Thrombus aspiration followed by direct stenting: a novel strategy of primary percutaneous coronary intervention in ST-segment elevation myocardial infarction. Results of the Polish-Italian-Hungarian RAndomized ThrombEctomy Trial (PIHRATE Trial).* Am Heart J, 2010. **160**(5): p. 966-72.

6. Kampinga, M.A., et al., *Thrombus Aspiration during Percutaneous coronary intervention in Acute non-ST-elevation myocardial infarction Study (TAPAS II)-Study design.* Neth Heart J, 2009. **17**(11): p. 409-13.

7. Diletti, R., et al., *ABSORB II randomized controlled trial: a clinical evaluation to compare the safety, efficacy, and performance of the Absorb everolimus-eluting bioresorbable vascular scaffold system against the XIENCE everolimus-eluting coronary stent system in the treatment of subjects with ischemic heart disease caused by de novo native coronary artery lesions: rationale and study design.* Am Heart J, 2012. **164**(5): p. 654-63.

8. Diletti, R., et al., *Clinical and intravascular imaging outcomes at 1 and 2 years after implantation of absorb everolimus eluting bioresorbable vascular scaffolds in small vessels. Late lumen enlargement: does bioresorption matter with small vessel size? Insight from the ABSORB cohort B trial.* Heart, 2013. **99**(2): p. 98-105.

9. Kajiya, T., et al., *Everolimus-eluting bioresorbable vascular scaffold (BVS) implantation in patients with ST-segment elevation myocardial infarction (STEMI).* EuroIntervention, 2013. **9**(4): p. 501-4.

10. Kubo, T., et al., *Multiple coronary lesion instability in patients with acute myocardial infarction as determined by optical coherence tomography.* Am J Cardiol, 2010. **105**(3): p. 318-22.

11. Yabushita, H., et al., *Characterization of human atherosclerosis by optical coherence tomography.* Circulation, 2002. **106**(13): p. 1640-5.

12. Tearney, G.J., et al., *Consensus standards for acquisition, measurement, and reporting of intravascular optical coherence tomography studies: a report from the International Working Group for Intravascular Optical Coherence Tomography Standardization and Validation.* J Am Coll Cardiol, 2012. **59**(12): p. 1058-72.

13. Gogas, B.D., et al., *Vascular response of the segments adjacent to the proximal and distal edges of the ABSORB everolimus-eluting bioresorbable vascular scaffold: 6-month and 1-year follow-up assessment: a virtual histology intravascular ultrasound study from the first-in-man ABSORB cohort B trial.* JACC Cardiovasc Interv, 2012. **5**(6): p. 656-65.

14. Gomez-Lara, J., et al., *Angiographic maximal luminal diameter and appropriate deployment of the everolimus-eluting bioresorbable vascular scaffold as assessed by optical coherence tomography: an ABSORB cohort B trial sub-study.* EuroIntervention, 2012. **8**(2): p. 214-24.

15. Abtahian, F. and I.K. Jang, *Optical coherence tomography: basics, current application and future potential.* Curr Opin Pharmacol, 2012. **12**(5): p. 583-91.

16. Rittersma, S.Z., et al., *Plaque instability frequently occurs days or weeks before occlusive coronary thrombosis: a pathological thrombectomy study in primary percutaneous coronary intervention.* Circulation, 2005. **111**(9): p. 1160-5.

17. Henriques de Gouveia, R., et al., *Sudden unexpected death in young adults. Discrepancies between initiation of acute plaque complications and the onset of acute coronary death.* Eur Heart J, 2002. **23**(18): p. 1433-40.

18. Murakami, T., et al., *Intracoronary aspiration thrombectomy for acute myocardial infarction.* Am J Cardiol, 1998. **82**(7): p. 839-44.
