# Supplementary material for: Thrombus characteristics evaluated by acute optical coherence tomography in ST elevation myocardial Infarction
Source: PLoS One. 2022 Apr 11;17(4):e0266634. doi: 10.1371/journal.pone.0266634 (PMC9000063; doi:10.1371/journal.pone.0266634)
Supplement: Attachment — Submitted filename: Response_to_Reviewers.docx [file pone.0266634.s003.docx]

Dear Editor and Reviewers,

Below are my rebutting comments. I hope the issues are addressed satisfactory.

The numbers in parantheses refers to line number in Manuscript with tracker changes.

1. Please ensure that your manuscript meets PLOS ONE's style requirements, including those for file naming. The PLOS ONE style templates can be found at

<https://journals.plos.org/plosone/s/file?id=wjVg/PLOSOne_formatting_sample_main_body.pdf>  and

<https://journals.plos.org/plosone/s/file?id=ba62/PLOSOne_formatting_sample_title_authors_affiliations.pdf>

Manuscript has been corrected to meet style requirements

2. Please remove the CONSORT flowchart and checklist, and the clinical trial protocol. These documents were erroneously requested upon submission since you selected 'clinical trial' as article type. However, we realize that your study is not directly related to the original clinical trial and therefore we processed it as a Research Article (where the clinical trial documentation is not needed). We apologize for the inconvenience.

In your manuscript, please clarify whether your original clinical trial has been published, providing a reference if applicable.

Orginal clinical trial manuscript has been submitted, not yet published.

Thank you for your attention to these requests.

3. Please include your full ethics statement in the ‘Methods’ section of your manuscript file. In your statement, please include the full name of the IRB or ethics committee who approved or waived your study, as well as whether or not you obtained informed written or verbal consent. If consent was waived for your study, please include this information in your statement as well.

The ethics statement has been moved from ‘Introduction’ to ‘Methods’ section (146-147)

4. In your Data Availability statement, you have not specified where the minimal data set underlying the results described in your manuscript can be found. PLOS defines a study's minimal data set as the underlying data used to reach the conclusions drawn in the manuscript and any additional data required to replicate the reported study findings in their entirety. All PLOS journals require that the minimal data set be made fully available. For more information about our data policy, please see <http://journals.plos.org/plosone/s/data-availability>.

Upon re-submitting your revised manuscript, please upload your study’s minimal underlying data set as either Supporting Information files or to a stable, public repository and include the relevant URLs, DOIs, or accession numbers within your revised cover letter. For a list of acceptable repositories, please see http://journals.plos.org/plosone/s/data-availability#loc-recommended-repositories. Any potentially identifying patient information must be fully anonymized.

Important: If there are ethical or legal restrictions to sharing your data publicly, please explain these restrictions in detail. Please see our guidelines for more information on what we consider unacceptable restrictions to publicly sharing data: <http://journals.plos.org/plosone/s/data-availability#loc-unacceptable-data-access-restrictions>. Note that it is not acceptable for the authors to be the sole named individuals responsible for ensuring data access.

We will update your Data Availability statement to reflect the information you provide in your cover letter.

A minimal data set has been uploaded as Supporting Information file.

[Note: HTML markup is below. Please do not edit.]

Reviewers' comments:

Reviewer's Responses to Questions

**Comments to the Author**

1. Is the manuscript technically sound, and do the data support the conclusions?

The manuscript must describe a technically sound piece of scientific research with data that supports the conclusions. Experiments must have been conducted rigorously, with appropriate controls, replication, and sample sizes. The conclusions must be drawn appropriately based on the data presented.

Reviewer #1: Yes

Reviewer #2: Yes

2. Has the statistical analysis been performed appropriately and rigorously?

Reviewer #1: Yes

Reviewer #2: Yes

3. Have the authors made all data underlying the findings in their manuscript fully available?

The [PLOS Data policy](http://www.plosone.org/static/policies.action#sharing) requires authors to make all data underlying the findings described in their manuscript fully available without restriction, with rare exception (please refer to the Data Availability Statement in the manuscript PDF file). The data should be provided as part of the manuscript or its supporting information, or deposited to a public repository. For example, in addition to summary statistics, the data points behind means, medians and variance measures should be available. If there are restrictions on publicly sharing data—e.g. participant privacy or use of data from a third party—those must be specified.

Reviewer #1: Yes

Reviewer #2: Yes

4. Is the manuscript presented in an intelligible fashion and written in standard English?

PLOS ONE does not copyedit accepted manuscripts, so the language in submitted articles must be clear, correct, and unambiguous. Any typographical or grammatical errors should be corrected at revision, so please note any specific errors here.

Reviewer #1: Yes

Reviewer #2: Yes

5. Review Comments to the Author

Please use the space provided to explain your answers to the questions above. You may also include additional comments for the author, including concerns about dual publication, research ethics, or publication ethics. (Please upload your review as an attachment if it exceeds 20,000 characters)

Reviewer #1: The authors sought to compare erythrocyte content and age of intracoronary thrombus assessed by histopathology and optical coherence tomography (OCT). The main conclusion of this study is that OCT could not differentiate between red and white thrombi, nor determine thrombus age.
Major concerns are:
1. As for such type of research, sample size is small.

I agree that the sample size is small. Unfortunately we had to terminate inclusion early, due to safety concerns of the Absorb© bioresorbable scaffold. However our reference studies have similar sample sizes. Kumar et al included 40 human cadavers and Silvain et al studied 45 thrombi. We think our study increases the knowledge about thrombi in ST elevation myocardial infarct, although it is not definite evidence.

2. The composition of the intracoronary thrombus depends on a distance from the vessel wall. Thrombi contain more fibrin in the superficial part, near the vessel wall whereas in the core they contain more erythrocytes. Thus, histopathology and OCT assessing not necessary the same part of thrombi were less likely to achieve a convergent result. This issue requires thorough discussion.

It is a good point that the remnants might be different from the aspirates, which we have mentioned in the discussion. We have elaborated on the issue under ‘Discussion’ (318-328).

3. Data regarding relationships between time of ischemia and thrombus composition in this sample size could be provided.

Ischemic time is found in Table. A more thorough analysis of subgroups has been added in ‘Results’ (259-263)

4. Independent determinants of histopathological content of thrombi versus OCT lightloss through the thrombus allow to compare their causative mechanisms

I understand this question as a request for analysis of determinants of thrombus contents in the vessesl wall, such as type of plaque, presence of lipid, calcification, thin cap atheroma or erosion. Unfortunatly most of the vessel wall is obscured by shadow from the thrombus, so that the characteristics of the vessel wall cannot be distinguished.

Reviewer #2: Dear Authors,
Your great clinical work is highly appreciated. Meticulously performed, great analysis.
Some major factor missing: time of chest pain (or ECG-diagnosis) to time of successful wiring.

You should correlate it with the OCT and histology findings.
Ischemic time is found in Table. A more thorough analysis of subgroups has been added in ‘Results’ (256-259)

Small issue: please, describe the details of OCT imaging (ml/s contrast, manual trigger?, how many runs were needed?).

The details has been elaborated in ‘Methods’ (162-164). Number of runs has been added to Table.

Your control of OCT automatic assessment with ImageJ is an outstanding concept.
Writing, typo, figures, referencing perfect.

However, I would not stress your "message" that these results might lead to change to current (pharmacologic? interventional?) approach to STEMI. Similarly, in the abstract, I would not this sentence: OCT to tailor anti-thrombotic treatment".

I agree that this message might be exaggerated. It was an attempt to relate the aim of the study to everyday clinical practice. The statement has been modified (92-93, 373-377 and 393-396).

I reckon that you (correctly) excluded patients with heavy calcification and complex lesions. I also disagree your opinion in the Discussion that you "have no reason to believe that our results would be different in more complex disease". Please give explanation.
Sincerely yours

I agree that we have no evidence showing thrombus formation behaves similarly in more complex and calcified lesions. However since our main finding is that OCT cannot differentiate thrombus content, we have no reason to believe that OCT is a better tool in such cases. Nevertheless, the statement has been cancelled (408-409).

Kind Regards,

Erlend Eriksen
